# Nurses' Experiences of the Quality of Family‐Centered Patient Counselling in Paediatric Delirium: A Qualitative Descriptive Study

**DOI:** 10.1002/nop2.70235

**Published:** 2025-04-28

**Authors:** Riitta Alanko, Miia Jansson, Tiina Saarenpää, Heli Kerimaa, Outi Peltoniemi, Miikka Tervonen, Tiina Lahtela, Tarja Pölkki

**Affiliations:** ^1^ Research Group of Health Sciences and Technology University of Oulu Oulu Finland; ^2^ RMIT University Melbourne Victoria Australia; ^3^ Pediatric Intensive Care Unit, Department of Children and Adolescents Oulu University Hospital Oulu Finland

**Keywords:** delirium, family‐centered care, nursing, patient counselling, prevention

## Abstract

**Aim:**

To describe nurses' experiences of the quality of family‐centred patient counselling in paediatric delirium.

**Design:**

A qualitative descriptive study.

**Methods:**

Semi‐structured interviews were conducted with 10 PICU nurses with experience in managing paediatric patients at high risk of developing delirium. The data were analysed inductively.

**Results:**

The study identified four main categories, 10 generic categories, and 34 specific subcategories within family‐centered patient counselling in paediatric delirium, offering a comprehensive framework to enhance counselling practices for families in paediatric delirium care.

**Conclusions:**

The study highlights areas to improve family‐centered patient counselling in paediatric delirium, particularly focusing on sufficiency, resources, and implementation. It emphasises the need for improved delirium‐related knowledge among healthcare providers. The study also suggests that digital services, such as websites and digital care pathways, can improve information access for families, enhancing the family‐centeredness of counselling.

**Patient or Public Contribution:**

None.

## 1 Background

Children in the paediatric intensive care unit (PICU) are at high risk of developing paediatric delirium (PD) due to underlying medical conditions, the effects of treatments and medications, and the stress of the PICU environment, which disrupts their sleep–wake cycles (Ista et al. 2023; Semple et al. 2022). PD is a form of acute brain dysfunction (American Psychiatric Association 2013) that affects 34%–71% of PICU admissions (Henao‐Castaño et al. 2023; Semple et al. 2022). It increases length of stay and duration of mechanical ventilation, increases mortality and healthcare costs, and decreases quality of life (Henao‐Castaño et al. 2023; Semple et al. 2022).

Family‐centered care (FCC) is a healthcare approach that prioritises collaboration between healthcare providers, patients, and their families to enhance care experiences and outcomes. It acknowledges the family as an integral part of the care team, ensuring their knowledge, values, and preferences are respected (Hill et al. 2018, 2019). Research has shown that FCC can lead to significant benefits, including a reduced incidence of delirium (Deng et al. 2020), lower anxiety and stress levels (Dahav and Sjöström‐Strand 2018), and an improved overall patient and family experience (Smith 2018). Furthermore, families express a strong desire to be actively involved in decision‐making regarding care and treatment (Fisk et al. 2022; Terp et al. 2021). However, despite this willingness, many families remain unaware of delirium management in highly technical PICUs (Pandhal and Van Der Wardt 2022; Smithburger et al. 2017).

Nurses play a crucial role in delirium management (Pandhal and Van Der Wardt 2022). Family presence and involvement in care are fundamental to patient‐centered counselling, which fosters a two‐way, interactive dialogue between patients and healthcare staff (Kääriäinen 2007). In nursing, high‐quality counselling integrates adequate resources with a goal‐oriented approach, ensuring that patients receive sufficient and effective guidance (Kääriäinen 2007). Both patient counselling and FCC emphasise communication, collaboration, and patient empowerment, sharing common objectives such as improving health outcomes, reducing anxiety, and providing personalised care. However, there is a gap in understanding how FCC can enhance the quality of counselling in the context of PD (Shrestha and Fick 2020).

In Finland, the national e‐health service, Health Village, provides essential information and support for patient care, as well as tools for healthcare professionals (Digital Care Pathways 2022; https://www.terveyskyla.fi/en/hubs). While FCC principles have been successfully integrated into nursing practices, a lack of specific counselling materials for PD remains. Supplementing verbal communication with written materials could improve the effectiveness and comprehensiveness of patient counselling.

Traditionally, the quality of counselling has been studied using quantitative methods (Kaakinen et al. 2017, 2020; Rajala et al. 2018). However, there is a need for more qualitative research to explore how FCC can improve patient counselling. This study aimed to describe PICU nurses' experiences with the quality of family‐centered patient counselling in PD, guided by the question: *How can the quality of patient counselling be improved to become more family‐centered in the context of pediatric delirium?*

## 2 Methods

### 2.1 Design

A qualitative descriptive design, guided by a phenomenological approach, was used to explore individuals' lived experiences through semi‐structured interviews with open‐ended questions (Polit and Beck 2017). In line with descriptive phenomenology, interpretation was minimised to focus on capturing and describing the essence of participants' experiences (Dowling 2007). The study was reported according to the Consolidated Criteria for Reporting Qualitative research checklist for qualitative studies (Tong et al. 2007).

### 2.2 Participants

The study was conducted in a single university hospital in Finland. The hospital has a mixed medical‐surgical PICU where different types of children and adolescents who require continuous monitoring are treated. Patients include surgical patients (e.g., back, skull, cleft lip/palate, and intestinal surgery) who need postoperative monitoring or pain management. Trauma, poisoning, burns, and neurological patients are also treated. The PICU is also the centre for initial treatment of severe diabetic ketoacidosis, renal and metabolic diseases, and severe infections. Length of stay varies from hours to months. The PICU has implemented a validated Cornell Assessment of Paediatric Delirium (CAPD) scale (Traube et al. 2014) to detect PD and a rehabilitation protocol to support early mobilisation (Deng et al. 2020) in children at high risk of developing delirium.

Participants were eligible if they were (1) registered nurses and (2) managed paediatric patients at high risk of developing delirium, while temporary nursing substitutes were excluded from the study. All respondents were asked the same questions in the same order. The interviews lasted between 29 and 49 min, during which the researcher had the opportunity to ask follow‐up questions if necessary.

### 2.3 Data Collection

All available PICU nurses (*N* = 45) were recruited by a contact person (T.L.), who informed volunteer participants about the study and asked for permission to pass on their contact details to the interviewers, which were conducted by master students (R.A., T.M.). Remote interviews were conducted individually using Microsoft Teams software between May and October 2022.

The theoretical framework was based on the hypothetical model of the quality of counselling (Kääriäinen 2007). In the model, the elements that contribute to the quality of counselling are identified as healthcare professional and patient‐related background factors, resources (including time, facilities, materials, and equipment), implementation (focusing on patient‐centeredness, interaction, and the overall atmosphere), sufficiency, and the effects of counselling.

The interviews were semi‐structured in nature. In line with descriptive phenomenology, open questions, such as *How can the content of patient counselling be developed to be more family‐centered in the context of PD*? and *How can digital services enhance the family‐centeredness of patient counselling in the context of PD*? were employed to elicit rich, authentic, and detailed accounts of participants' lived experiences.

The interview guide was reviewed and pilot‐tested by clinicians before starting the interviews. The appropriateness of the final sample size was assessed throughout the interviews. Interviews were audio‐recorded and then transcribed verbatim by the first author.

### 2.4 Data Analysis

The data were analysed inductively by the first author (Kyngäs et al. 2020). The unit of analysis (phrases) was defined before the start of the analysis process. Hidden content such as sighs, pitch changes, and pauses were excluded from the analysis of the data.

First, the data were read several times. Second, the transcribed text was divided into original expressions and reduced expressions. Third, similar expressions were grouped into subcategories by the first, second, and last author. Fourth, similar subcategories were grouped into general categories, which were further combined into four main categories. Finally, each main category was named using the theoretical framework and content‐characteristic words.

### 2.5 Ethical Considerations

The study was approved by the relevant academic centre (Decision No: 86/2021) and reviewed by the local ethics committee in autumn 2022 (EETTMK: 86/2021). Written informed consent was obtained from nurses prior to inclusion in the study to ensure that participation was voluntary (World Medical Association 2013). Respondents were not affiliated with the researchers. The processing, backup, and security of the research data were systematically ensured in accordance with the Data Protection Act (1050/2018) and the EU General Data Protection Regulation (EU 679/2016).

### 2.6 Rigour

Rigour in phenomenological nursing research was demonstrated, ensuring credibility, dependability, confirmability, authenticity, and transferability (Kyngäs et al. 2020). To ensure credibility, the interviews were audio‐recorded and transcribed verbatim to ensure that each response was fully and accurately recorded for data analysis. To ensure dependability, the research process was documented carefully (Table 1), coded, and reviewed by other researchers (M.J., T.P.). To ensure confirmability, direct quotes were included to ground interpretations in the data. To support authenticity, multiple citations have been presented to demonstrate the authentic experience of the participants. To ensure transferability, the sample selection and the data analysis process were explained in detail, and the findings were presented without any comments. Data saturation was monitored by two researchers (R.A. and T.P.) and was deemed achieved when further interviews no longer revealed new insights into the phenomenon being studied (Kyngäs et al. 2020).

**TABLE 1:** Examples of citing, data analysis, and categorisation of the data.

Main category	Generic category	Subcategory	Sample citations
Resources of family‐centered patient counselling in PD	Counselling methods and materials	Written materials	*…but the written material supports the ability to return to the topic* (108)
		Health information technologies	*in order to make it* (patient counselling) *more family‐centered, information access should be improved, perhaps in writing, either on paper or through digital care pathways* (108)

## 3 Results

The study was conducted with 10 PICU nurses. Most of the nurses were female, aged between 23 and 61 years. The study identified key areas for developing more family‐centered patient counselling in the context of PD. Four main categories emerged: sufficiency, resources, implementation, and healthcare professional‐related background factors. These categories were further broken down into 10 generic categories and 34 specific subcategories, which provide a comprehensive framework for improving family‐centered patient counselling in PD care (Table 2).

**TABLE 2:** The main, generic, and subcategories of the quality of family‐centered patient counselling in paediatric delirium (PD).

Main categories	Generic categories	Subcategories
Sufficiency of family‐centered patient counselling in PD	Providing comprehensive information about PD	Risk factors, symptoms, and prevention of PD
		Medical procedures
	Fostering active family involvement in care	Early rehabilitation
		Care and recovery
Resources of family‐centered patient counselling in PD	Optimising the timing and amount of information provided	Splitting information
		Level of details
		Clear communication using plain language and avoiding jargon
	Fostering a family‐centered counselling environment	Familiar objects
		Unresting facilities and multi‐patient rooms
		Quiet spaces
		Presence of siblings and grandparents
	Enhancing counselling methods and materials	Reliable health information
		Written materials
		Health information technologies
		Digital care pathways
		Two‐way feedback mechanism using information technologies
Implementation of family‐centered patient counselling in PD	Patient‐centeredness	Clear information about the daily routine of the PICU
	Emotional support	Reduce the anxiety among family members
		Mental preparation of the parents
		Assessment and treatment of the emotional state of parents
		Honest but discreet communication
		Family involvement in care without judgement
	Interprofessional collaboration	Out‐of‐hospital services for families
		Social, family, and crisis workers
		Interprofessional bedside screening tool
		Clinical pathway for screening/treatment of paediatric delirium
Healthcare professional‐related background factors in PD	Enhancing knowledge related to delirium	Recognition and management of PD
		Distinguish PD from other symptoms
		Lack of knowledge of PD
	Improving skills in managing delirium	Irregular use of bedside screening tools
		Arrival screening
		Use of screening tools
		Continuous nursing education
		Nursing curricula

### 3.1 Sufficiency of Family‐Centered Patient Counselling in Paediatric Delirium

The sufficiency of family‐centered patient counselling plays a crucial role in early recognition, risk‐based prevention, early identification, and proper management of PD. To improve the quality of patient counselling in the context of PD, it is vital to provide comprehensive information about PD and actively involve families in the care process.

#### 3.1.1 Providing Comprehensive Information About PD

According to the interviewees, families should be well‐informed about risk factors, symptoms, and prevention strategies for PD. One participant emphasised the importance of informing parents about medical procedures, stating, *Parents should be told everything—why it is being done, how it is done, where it is done, and what it means in practice* (110). This approach keeps parents informed and involved, reducing anxiety and stress levels.

#### 3.1.2 Fostering Active Family Involvement in Care

In addition to offering comprehensive knowledge, fostering active family involvement in care is equally crucial. Interviewees noted that families should be informed about the importance of early rehabilitation and encouraged to actively participate in their child's care and recovery. For example, one nurse stated: *…upon arrival at the ward, inform the parents that we begin early rehabilitation in the ICU … this way, parents can be involved from the start* (104). By involving families early in the process, they become active participants in the rehabilitation journey, which can improve the family's sense of empowerment and support.

### 3.2 Resources of Family‐Centered Patient Counselling in Paediatric Delirium

The admission of a child to intensive care is often an unexpected and profound event for families, placing considerable emotional strain on them. Alongside the challenges of PICU admission, PD significantly heightens the need for families to receive timely, comprehensive information and support. To improve the quality of patient counselling in the context of PD, it is crucial to optimise the timing and amount of information provided, foster a family‐centered counselling environment, and enhance counselling methods and materials. With the increasing opportunities provided by digitalisation, these elements will become even more critical in shaping the future of FCC in PD.

#### 3.2.1 Optimising the Timing and Amount of Information Provided

According to the interviewees, information should be provided in stages and in a timely manner, as parents may have limited capacity to fully comprehend all the information at once. One nurse explained, *Informing and telling things in advance and then giving information every day and sort of going through the situation* (106). Additionally, the level of detail should be tailored to individual needs, ensuring clarity without overwhelming the family. As one nurse stated, *…by no means all the possible risks, because then the parents will then be afraid of them, and it may be that life will be limited to looking for those risks* (102). Overall, communication should be as clear and straightforward as possible, using simple, plain language and avoiding technical jargon to ensure that families fully understand the information being shared.

#### 3.2.2 Fostering a Family‐Centered Counselling Environment

According to the interviewees, parents can help create a safe environment for their child by bringing familiar objects from home that are important to the child when admitted to the PICU. However, the counselling environment was considered inadequate due to the unrestful facilities and multi‐patient rooms. One interviewee noted, *The current situation is such that the family‐centered approaches cannot be implemented in the best possible way* (110). Interviewees emphasised that family‐centered approaches require quiet spaces, opportunities for families to calm down, and the ability to be with the child as much as possible, including at night. Additionally, the presence of siblings and grandparents should be allowed to support the family during this time.

#### 3.2.3 Enhancing Counselling Methods and Materials

In the PICU setting, parents may find it challenging to fully comprehend all the new information presented to them. Providing written materials can help make the information easier to internalise and allow parents to revisit the topic when needed. As one interviewee stated, …*but the written material supports the ability to return to the topic* (108). However, there is a lack of counselling materials in the PICU. The availability of written materials could complement verbal information and support nurses in effectively implementing patient counselling.

According to the interviewees, digital services can significantly enhance the family‐centeredness of patient counselling in the context of PD by providing reliable and accessible health information. Health information technologies, such as websites and digital care pathways, are seen as vital tools for improving access to crucial information. One interviewee noted that improving access to information, whether in written form or via digital platforms, could make counselling more family‐centered: *To make patient counselling more family‐centered, access to information should be improved, perhaps in written form, either on paper or through digital care pathways* (108).

Interviewees suggested that new digital care pathways should be integrated into Health Village, a Finnish public online service, to support families and address the needs of paediatric intensive care. However, a challenge highlighted by interviewees is the wide range of illnesses and injuries in the PICU, which makes it difficult to create a one‐size‐fits‐all digital care pathway for every possible condition.

There is a clear need for a two‐way feedback mechanism. Parents want the opportunity to provide feedback on their experience in the PICU, while nurses also seek regular feedback from families to improve their practice. According to the nurses, transparent and anonymous feedback could be facilitated through desktop computers, laptops, or mobile devices using a pre‐designed QR code for easy access and submission.

### 3.3 Implementation of Family‐Centered Patient Counselling in Paediatric Delirium

In the PICU, there is a certain rhythm to the day, and nurses believe it would be beneficial to involve both the child and parents in this daily routine. Additionally, the need for emotional support becomes more pronounced in abnormal circumstances. When a child falls ill, families face numerous challenges and require a good listener, a source of comfort, and an empathetic nurse. Providing emotional support demands strong interpersonal skills and knowledge from the nurse. To enhance the implementation of family‐centred patient counselling in the context of PD, it is essential to focus on patient‐centred care, emotional support, and interprofessional collaboration. However, according to the interviewees, busyness and staff shortages were identified as significant barriers to effectively implementing FCC.

#### 3.3.1 Patient‐Centeredness

According to the interviewees, both the child and the family should be informed about the daily routine of the PICU. When parents are aware of the routine, they can plan their own day more effectively and anticipate what will happen and when. Additionally, parents are considered the child's best experts and can contribute to establishing a familiar daily routine for the child. As one interviewee noted, *Following the daily routine … I could ask the parents during the shift what time the child goes to sleep and try to align the evening activities, such as dimming the lights, with that time* (105).

#### 3.3.2 Emotional Support

According to the interviewees, parents often seek emotional support, and increasing awareness of the clinical presentation of delirium could help reduce anxiety among family members. One interviewee stated, *Parental anxiety can be alleviated by explaining the symptoms of delirium and how it appears in the child* (105). Additionally, mental preparation for parents was considered essential. Nurses must assess and address the parents' emotional state, providing support in a way that is both honest and discreet. The goal is not to create further embarrassment or anxiety when sharing information. As one nurse explained, *Information should be provided honestly, but in a way that does not cause too much fear and panic* (110).

Nurses should recognise the diverse levels of parental involvement in a child's care, acknowledging that some parents may feel guilty about limited time with their child, while others may prefer minimal involvement. Conversely, some parents may wish to take on all caregiving responsibilities, even when it may not be in the child's best interest. Understanding and respecting these differences is crucial in FCC. One nurse highlighted the importance of aligning care with the child's routine, stating, *Following the daily rhythm … I might ask the parents during the shift what time the child goes home to sleep and try to do the evening activities and dim the lights at that time* (105).

#### 3.3.3 Interprofessional Collaboration

According to the interviewees, nurses often spend a significant amount of time addressing acute situations in the early stages of care, which can result in parents being overlooked. As the treatment becomes more prolonged and complex, access to out‐of‐hospital care can provide much‐needed support for families. One interviewee explained, *…these things are left there to weigh on your shoulders … you should go somewhere to talk and unpack once in a while* (101).

Nurses emphasised the importance of understanding the family's overall situation in order to provide holistic support. While social and family workers were available in the past, they are no longer accessible within the organisation. However, crisis workers are available, and their use is particularly recommended during the acute phase of care. One interviewee explained, *…he work is so acute in the early stages, and the parents are in a crisis situation at the very beginning. Sometimes, when parents are offered even basic discussion help, they may feel they haven't yet internalized the event. It's important to have a lower threshold for access to crisis workers* (105).

PICU nurses use the CAPD bedside screening tool to detect delirium in patients. If the screen is positive (CAPD ≥ 9), the PICU clinical team, including the paediatric intensivist and physiotherapist, should assess the patient. However, according to the interviewees, there is a lack of a consistent clinical pathway for screening and treatment of children at high risk of developing delirium. One interviewee noted, *…we still lack consistent protocols on how to proceed with it* (110).

### 3.4 Healthcare Professional‐Related Background Factors in Paediatric Delirium

The availability of resources for family guidance presents challenges for organisations, particularly those struggling to attract a skilled workforce. This issue also highlights the difficulties inherent in patient counselling and the critical skills nurses must develop to effectively manage counselling in a way that meets families' needs. To strengthen healthcare professional‐related background factors in the context of PD, it is essential to focus on enhancing knowledge related to delirium and improving skills in managing delirium.

#### 3.4.1 Enhancing Knowledge Related to Delirium

Interviewees highlighted the challenges of recognising and managing PD, as its symptoms can overlap with pain and withdrawal symptoms. The varied clinical presentation, particularly in children with developmental disabilities, adds to the complexity. A significant barrier to supporting families is the lack of knowledge about PD among nurses. As one interviewee stated, *Not all nurses have much information about pediatric delirium; it's still in its infancy … You can't support the parents if you don't know anything about it yourself*. This underscores the urgent need for increased education and awareness among healthcare professionals to enhance support for both children and their families.

#### 3.4.2 Improving Skills in Managing Delirium

Interviewees noted that the use of the CAPD bedside screening tool is inconsistent, with screening often delayed upon a child's admission to the PICU. A lack of familiarity with the tool among nurses further hinders early detection, leading to calls for additional training. Furthermore, interviewees emphasised the importance of incorporating delirium‐related education into nursing curricula to equip future nurses with the necessary skills to recognise and manage paediatric delirium effectively.

## 4 Discussion

The study examined how to make patient counselling more family‐centered in the context of PD, identifying four key areas for improvement: sufficiency, resources, implementation, and healthcare professional‐related background factors. It highlighted that digital services could enhance family‐centered patient counselling by providing accessible and reliable health information. Health information technologies, such as websites and digital care pathways, can improve access to essential information for families.

The sufficiency of family‐centered patient counselling in PD emphasises providing clear, adequate information and actively involving families. It underscores the importance of family‐based delirium management strategies, aligning with previous research that highlights families' critical role in the early identification and management of PD (Pandhal and Van Der Wardt 2022; Shrestha and Fick 2020). Parents also express a strong desire to participate in decision‐making regarding their child's care (Fisk et al. 2022; Terp et al. 2021; Richards et al. 2017).

In general, person‐centered and equitable collaboration between parents and nursing staff positively impacts parental satisfaction (Terp et al. 2021; Phiri et al. 2020). However, parental involvement should be tailored to their willingness and readiness to participate in decision‐making (Terp et al. 2021). To facilitate meaningful engagement, PICUs should establish clear guidelines on the extent of parental participation and the roles they can assume in their child's care and decision‐making process.

The resources for family‐centered patient counselling in PD focus on delivering information at the right time, within a supportive family‐centered environment, and through appropriate methods and materials. These resources emphasise both the timing and amount of information shared, as well as the overall counselling environment. Consistent with Fisk et al. (2022), nurses should assess what health information and the level of detail each family requires to provide comprehensive, individualised counselling.

The physical and cultural environment plays a crucial role in improving both patient (Deng et al. 2020) and family experiences (Hill et al. 2019). Interventions such as light therapy, earplugs, and eye masks can enhance sleep quality, while well‐designed patient rooms, waiting areas, and overall unit layouts contribute to preserving dignity and reducing stress. However, interviewees noted that the current counselling environment was inadequate due to unrestful facilities and multi‐patient rooms. The highly technological, unpredictable, and stressful nature of the PICU itself can serve as a barrier to FCC (Hill et al. 2019).

To address these challenges, parents require structured guidance from nursing staff to facilitate active participation in their critically ill child's care at the bedside (Fisk et al. 2022). Providing clear, accessible information within a supportive environment can help empower families and enhance their ability to engage meaningfully in the care process.

The interviewees highlighted the potential of digital services to enhance family‐centered patient counselling in the context of PD by providing accessible and reliable health information. Health information technologies, such as websites and digital care pathways, are seen as essential tools for improving access to vital information. One interviewee noted that improving access to information—whether through written materials or digital platforms—could make counselling more family‐centered. For example, asynchronous video‐based educational interventions have been shown to significantly improve both family members' and healthcare staff's understanding of delirium detection, prevention, and management (Kamdar et al. 2022; Krewulak et al. 2020).

The implementation of family‐centered patient counselling in PD emphasises the importance of patient‐centeredness, emotional support, and fostering interprofessional collaboration. The findings highlighted that family, social, and crisis workers could offer more holistic support during times of crisis, ensuring a more integrated and supportive approach for families. This collaborative model aims to enhance the overall care experience, addressing both the emotional and informational needs of the family while managing paediatric delirium effectively.

Both enhancing knowledge of delirium and improving skills in its management are essential for better recognising, managing, and providing FCC for children experiencing delirium. In the PICU, nurses must be familiar with the symptoms, risk factors, and prevention strategies for PD, as well as the use of screening tools like the CAPD scale. However, despite the implementation of the CAPD scale and a rehabilitation protocol to detect PD and support early mobility, there is still a lack of knowledge regarding the clinical pathway for screening and treatment of PD, as noted in previous literature (Ragheb et al. 2023). This knowledge gap hinders effective delirium management and underscores the need for improved education and protocols in the PICU setting.

### 4.1 Strengths and Limitations

This study has several limitations related to its design, methods, and analysis. First, the interviews were conducted in a single PICU, meaning the findings may not be generalisable to other settings due to potential differences in organisational policies or cultures. However, the majority of the findings are consistent with existing literature, which lends support to their validity. Second, due to time constraints, the transcripts were not shared with the interviewees for review, though direct quotes were included to enhance credibility and ensure the accuracy of the data. Despite the small sample size, data saturation was achieved, indicating that the study gathered sufficient information to address the research question effectively.

### 4.2 Recommendations for Further Research

A similar qualitative study involving parents and families should be conducted to gain additional insights into their experiences and perspectives. The findings from both studies could then be integrated and tested quantitatively to confirm a midrange theory. Furthermore, additional research is needed to evaluate the effectiveness of family‐based delirium management strategies in paediatric delirium. It is also crucial to develop protocols and guidelines that promote FCC, ensuring best practices and improving care quality in the PICU. Our findings revealed that nurses were often unaware of the implications of FCC and its significant impact on nursing practices, highlighting the need for further education and training in this area.

## 5 Conclusion

The study explored improving family‐centered patient counselling in PD, identifying key areas for enhancement: sufficiency (providing adequate information and involving families in care decisions), resources (timely delivery of information in a family‐centered environment using appropriate methods), and implementation (focusing on patient‐centered care, emotional support, and interprofessional collaboration). It also highlighted the importance of healthcare professional‐related background factors, stressing the need for better delirium knowledge and skills among healthcare providers. Additionally, the study pointed out that digital services could enhance family‐centered patient counselling by offering accessible, reliable health information through technologies like websites and digital care pathways, improving families' access to essential information.

## Author Contributions

M.J., O.P., M.T., T.P.: design. R.A., T.S., T.L.: data collection. R.A., H.K., M.J., T.P.: analysis. R.A., M.J., T.S., H.K., O.P., M.T., T.L., T.P.: writing the report.

## Ethics Statement

The study was approved by the relevant academic centre (Decision No: 86/2021) and reviewed by the local ethics committee in autumn 2022 (EETTMK: 86/2021).

## Conflicts of Interest

The authors declare no conflicts of interest.

## Data Availability

Authors elect not to share data due to ethical restrictions.
